# Parliament and Publications

**Published:** 1911-05-06

**Authors:** 


### PARLIAMENT AND PUBLICATIONS.

### EYE DISEASES AND ALIENS.

Replying to a question asked by Mr. Hunt, the Home Secretary stated that it was not a fact that the medical inspection under the Aliens Act had been in any way relaxed. The reasons for the reduction in the number of rejections for trachoma in 1910 as compared with 1909 are, firstly, that in consequence, no doubt, of the rejections in previous years, the number of aliens suffering from trachoma who arrived on immigrant ships decreased ; and, secondly, that the figures for 1909 were swollen through the arrival in special circumstances of numbers of Armenians and Syrians who were suffering from the disease.

### A PUBLIC VACCINATOR'S FEES.

Mr. Lansbury asked the President of the Local Government Board if a public vaccinator in Bermondsey pays 2s. to the parent of every child taken to him for vaccination; and if this were the case, would he consider the advisability of recommending the Bermondsey Board of Guardians to reduce the fees paid to public vaccinators in that union? Mr. Burns replied that one of the public vaccinators in Bermondsey is also a teacher of vaccination, authorised to give certificates of proficiency in vaccination to persons who may afterwards become public vaccinators. He makes some payments to the mothers of children who bring them for vaccination to the classes which he holds for the instruction of students. Mr. Burns does not see sufficient reason for recommending a reduction of the fees paid by the Guardians to the public vaccinator.

### BUBONIC PLAGUE IN INDIA.

Referring to a question, Mr. Montagu stated that the number of deaths due to bubonic plague in the United Provinces of Agra and Oudh in February was 43,508, and in March 95,884. Every effort is being made to stamp out the disease; reliance is mainly placed upon evacuation of dwellings and inoculation, together with rat destruction and disinfection of houses.

